# Development of a hybrid sleep and physical activity improvement intervention for adults with osteoarthritis-related pain and sleep disturbance: a focus group study with potential users

**DOI:** 10.1177/20494637211026049

**Published:** 2021-06-25

**Authors:** Daniel Whibley, Kevin Stelfox, Alasdair L Henry, Nicole KY Tang, Anna L Kratz

**Affiliations:** 1 Epidemiology Group, School of Medicine, Medical Sciences and Nutrition, University of Aberdeen, Aberdeen, UK; 2 Department of Physical Medicine & Rehabilitation, University of Michigan, Ann Arbor, MI, USA; 3 Department of Anesthesiology, Chronic Pain & Fatigue Research Center, University of Michigan, Ann Arbor, MI, USA; 4 School of Education, University of Aberdeen, Aberdeen, UK; 5 Big Health Inc., San Francisco, CA, USA; 6 Big Health Inc., London, UK; 7 Sleep and Circadian Neuroscience Institute, Nuffield Department of Clinical Neurosciences, University of Oxford, Oxford, UK; 8 Department of Psychology, University of Warwick, Coventry, UK

**Keywords:** Osteoarthritis, sleep, physical activity, intervention development, focus group

## Abstract

**Objective:**

Suboptimal sleep and physical activity are common among people living with osteoarthritis (OA) and simultaneous improvements in both may have a beneficial impact on pain. This study aimed to gather perspectives of people living with OA on important aspects to incorporate in a hybrid sleep and physical activity improvement intervention for OA pain management.

**Design:**

Qualitative study using two rounds of two focus groups.

**Setting and participants:**

Focus groups were conducted with adults living with OA-related chronic pain and sleep disturbances. Eighteen people attended focus groups in January 2020 and, of these, 16 attended subsequent focus groups in February 2020.

**Methods:**

Discussion at the first round of focus groups informed generation of prototype intervention materials that were shared, discussed and refined at the second round of focus groups. Thematic analysis was used to identify themes and sub-themes from the data.

**Results:**

Three themes, each with three sub-themes, were identified: facilitators of engagement with the intervention (sub-themes: motivational language, accountability and education); barriers to engagement (sub-themes: suboptimal interaction with healthcare practitioners, recording behaviour as burdensome/disruptive and uncertainty about technique) and characteristics of a physical activity intervention component (sub-themes: tailored, sustainable and supported).

**Conclusion:**

We have identified important aspects to incorporate into the design and delivery of a hybrid sleep and physical activity improvement intervention for OA pain management. Insights will be incorporated into intervention materials and protocols, with feasibility and acceptability assessed in a future study.

## Introduction

Osteoarthritis (OA) is very common, affecting an estimated 30.8 million adults in the United States^1^ and 8.5 million in the United Kingdom.^2^ Pain is a cardinal symptom and, in the absence of a cure for OA, is experienced as chronic and fluctuating.^3^ Qualitative research has revealed a perception among those with OA that increasing activity increases pain.^4^ Activity-induced pain can be a substantial barrier to physical activity (any movement resulting in energy expenditure) and engagement in exercise (planned and structured physical activity aimed at improving fitness).^5^ This is unfortunate given possible analgesic benefits of regular exercise for OA-related pain in the long term reflected in its centrality in OA self-management recommendations.^6–8^ Identifying methods to support adoption of physical activity/exercise behaviours despite pain exacerbation in the acute period is therefore critical to support effective behaviour change. Interventions that support increasing physical activity while concurrently addressing other troublesome OA symptoms may be particularly attractive.

In addition to pain, most people living with OA also experience problems with sleep,^9,10^ and poor sleep quality and suboptimal sleep duration (too short or too long) have been associated with greater pain intensity.^9,11^ Given consistent research findings from longitudinal study that support a bidirectional association between sleep and pain, and evidence which indicates that sleep has a stronger impact on pain rather than vice versa,^12^ sleep optimization has been identified as a potentially important feature of OA pain management.^10^ Self-reported restless sleep has been associated with lower levels of objectively measured physical activity among adults with knee OA,^13^ and in more general populations, evidence from prospective study indicates a bidirectional association between measures of sleep and physical activity.^14,15^ This bidirectional association may be exploited in the early stages of adoption of physical activity and exercise programmes for people living with OA – if sleep can be improved, physical activity engagement may increase; as physical activity levels increase, sleep may improve. Indeed, it is possible that a hybrid intervention that simultaneously optimizes sleep *and* physical activity may provide greater analgesia than either component in isolation. This proposition is supported by observational evidence that indicates that perceived sleep quality and objectively measured sleep duration are important regulatory factors in the physical activity–pain association.^16–19^

To date, hybrid interventions for chronic pain that have included a sleep component have incorporated cognitive behavioural therapy for insomnia (CBT-I), the guideline recommended treatment for insomnia,^20^ with promising results.^21–25^ These hybrid interventions have all delivered CBT-I in-person. However, CBT-I interventions delivered digitally (dCBT-I), for example, online websites or mobile phone apps, have also demonstrated efficacy.^26–28^ Digital delivery of CBT-I content (including evidence-based cognitive and behavioural techniques, sleep hygiene education and relaxation exercises) may be particularly attractive given its flexibility of use, scalability, increased accessibility for hard to reach groups and suitability during periods when social distancing may be required. In addition, digital delivery may overcome many of the barriers associated with accessing in-person CBT-I, including limited numbers and poor geographical distribution of trained providers.^29^ *Sleepio* is one such fully automated (i.e. requiring no human input) digitally delivered programme that provides evidence-based dCBT-I across six sessions. *Sleepio* is supported by 12 randomized controlled trials that document significant improvements in poor sleep and insomnia symptoms in a range of populations.^30–41^ The characteristics of the programme make it well-suited as a sleep improvement component around which a physical activity or exercise programme for OA pain management could be developed.

Although some established exercise programmes for OA-related pain include short modules on sleep hygiene, sleep hygiene in isolation is minimally effective at improving sleep^42^ and a hybrid intervention that has a specific aim to simultaneously optimize sleep and physical activity has not yet been developed. Also, existing hybrid psychological approaches for sleep management for those with chronic pain have yet to make use of dCBT-I. We, therefore, plan to develop a hybrid intervention for OA-related pain management that aims to improve sleep using an established dCBT-I intervention (*Sleepio*) while concurrently targeting physical activity behaviour. To ensure acceptability and likelihood of intervention uptake, dissemination and sustainability, it is important that potential users are involved in the development process.^43^ The aim of this focus group study was to involve people living with OA-related pain and sleep disturbance in the development of a hybrid sleep and physical activity improvement intervention prior to engaging in feasibility testing.

## Methods

A study protocol (unregistered) was submitted to the Institutional Review Boards of the University of Michigan Medical School and was classified as exempt from full review under the condition that the study was restricted to focus groups and procedures were in place to ensure recordings prevented participant identification. The protocol was adhered to and the study completed in accordance with the Helsinki Declaration as revised in 2013. This report follows guidance described in Consolidated Criteria for Reporting Qualitative Studies.^44^

Four focus groups were conducted with people with OA-related pain and sleep disturbances. Two focus groups were conducted in January 2020 and two in February 2020 with the same participants. We aimed to recruit 16 participants (eight for each focus group). Anticipating some attrition between time of recruitment and the date of the first round of focus groups, ten participants were recruited to each group. Number of participants and rounds of groups were predetermined based on a balance between expected interactions between group members, data quality and resource constraints and not anticipated data saturation.^45^ It was acknowledged among the research team that if insufficient meaning was derived from collected data, additional focus groups with different participants would be considered.

Focus groups were conducted at a research facility at the University of Michigan, Ann Arbor, lasting 2 hours each. They were facilitated by two research team members (DW and an assistant).

### Participant selection

An advertisement was posted on the University of Michigan Clinical Studies Research Registry website. Potential participants were identified using purposive sampling to include a broad range of demographics (age, gender and race/ethnicity). Respondents were screened via telephone and were considered eligible if they were aged ⩾ 18 years with self-reported physician-diagnosed OA, any level of troublesome pain (for at least 3 days/week for the last 3 months) and sleep disturbance (for at least 3 days/week for the last 3 months), including difficulty getting to sleep, staying asleep, waking earlier than desired or waking feeling unrefreshed. Exclusion criteria were having a systemic, inflammatory musculoskeletal disorder, malignancy, a neurological movement disorder or if they had been told by a healthcare practitioner to avoid exercise for any reason. After confirming eligibility, participants were emailed an information sheet and informed consent form. On attending the first focus group, each participant was greeted by DW and taken to a private room where a hard copy informed consent form was signed and dated. Participants were compensated US$40 for attendance at each focus group.

### Focus group facilitation

All groups were facilitated by DW, a White cis-gender man and UK-registered physiotherapist with clinical experience delivering physical activity programmes for people living with long-term conditions. At the time of the study, DW was a postdoctoral research fellow and had completed the 6-week *Sleepio* course. DW made initial telephone contact with participants, followed this up with an email and, with permission, emailed/called a week before each group to remind them about the session. At the first in-person meeting, DW introduced himself and his background, explained the current project and the broader research programme within which it was situated. Participants were informed that focus group interactions would have no impact on their healthcare. Although DW had completed formal postgraduate training in qualitative research, as this was his first experience conducting a focus group study, he was supervised and guided by ALK throughout. ALK is a White cis-gender woman with extensive experience of qualitative research, including conducting focus group studies. A co-facilitator was present at all focus groups who ensured recording devices operated as expected, assisted participants when necessary and keep field notes of any non-verbal events. Researchers from Big Health Inc. (the company responsible for *Sleepio*) played no role in the focus groups.

### Focus group materials

**Figure 1.**
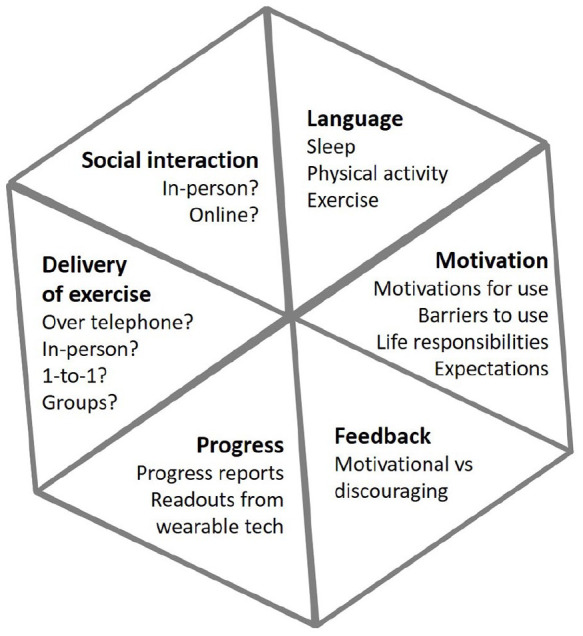
Pre-specified domains for focus group discussions.

*Round 1, January 2020*: The concept of a hybrid sleep and physical activity improvement intervention was described. An introductory video about *Sleepio* was played and it was explained that we wanted to develop an exercise or physical activity intervention around this 6-week course of dCBT-I. It was explained that we were primarily interested in gathering perspectives on six different aspects of the design and delivery of the intervention (Figure 1), and that after the first round of focus groups, we would use discussion content to generate prototype materials that we would present at the second round of focus groups to facilitate continued discussion. The six aspects of study design and a semi-structured topic guide for each aspect (see Supplement 1) were informed by a Participatory Action Research process based on theories of Behaviour Change and Persuasive Technology.^46^

*Round 2, February 2020*: A slideshow was prepared that summarized discussions from Round 1. Areas of commonality and divergence between the two groups were shared, acting as foci for discussion. How discussions from Round 1 informed development of intervention prototype materials was described, and a range of options were presented. These included possible names for the intervention, an intervention timeline, workbooks, different pain questionnaire options and a range of wrist-worn accelerometers that could be used to measure movement.

### Data analysis

Focus groups were audio recorded, transcribed verbatim by an external company and checked for accuracy against original audio recordings. Data were analysed using thematic analysis within a content analysis theoretical framework.^47^ Deductive and inductive approaches were incorporated to explore specific issues while leaving room for participants’ experiences to inform development of codes and themes.^48^ Transcripts from Round 1 focus groups were examined by DW, with preliminary themes and sub-themes identified, reviewed by ALK against Round 1 transcripts. Findings were shared at Round 2 focus groups. Once transcriptions of all four focus groups were complete, data were stored and analysed in NVivo. Two researchers (DW and KS) independently listened to recordings and read transcripts multiple times, identifying meaningful sections. These were compared and discussed, and a shared understanding of the data was agreed upon. Transcript content was then coded, reviewed and developed further to form sub-themes. Sub-themes were then grouped into overarching themes. The coding process and development of themes was iterative, with coders reviewing data and moving backwards and forwards between initial coding, second-level coding, sub-themes and themes to represent focus group perspectives. On completion of a draft manuscript, all focus group participants were given the opportunity to review and comment on the findings.

## Results

**Figure 2.**
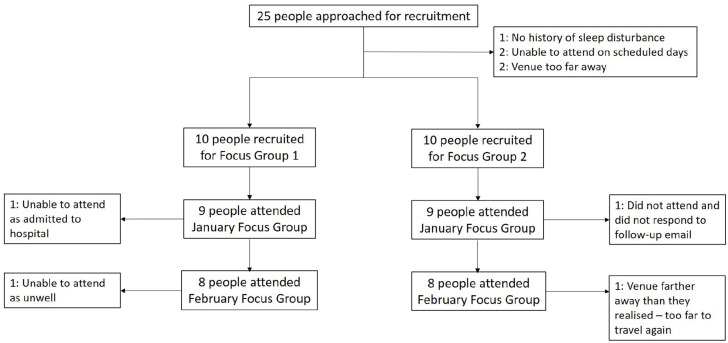
Flow chart of participants through the study.

Of 25 people contacted in response to their interest in the study, 20 were initially recruited. Of these, 18 attended Round 1 focus groups (nine in each group) and 16 attended Round 2 (eight in each group). Figure 2 illustrates the flow of participants through the study. Of 18 people who attended at least one group, median age was 67.5 (range = 47–76), 78% were women, 83% White and 17% Black. The majority reported widespread OA pain that affected the low back, upper and lower limbs (median duration of OA: 17 years). Discussions around pre-specified domains (Figure 1) at Round 1 and prototype intervention materials at Round 2 were used to identify sub-themes within three major themes of: (1) facilitators, (2) barriers to engagement with the intervention and (3) characteristics of the physical activity component (Tables 1–3; additional participant quotes provided in Supplement 2).

**Table 1.** Theme 1 – facilitators of engagement with the intervention.

Sub-theme	Participant quote
Motivational language	When I was a kid, when were in P.E. class and we did something wrong we had to run an extra lap or work out extra was like punishment, but it should be a gift like health enhancement activities or something . . . It’s got a bad taste in my mouth, ‘exercise’ [Participant 3, FG 1, round 1]
	I like the ‘move, recover and progress’ because in fitness, you have to take time down to recover to let your muscles recover, you can’t keep doing the same thing . . . I like ‘move recover, progress’ because it shows forward movement [Participant 8, FG2, Round 1]
	If you’re in a sport in school and you have a coach, they’re as bigger cheerleader to you as what your football cheerleading team would be. So your coach is kind of . . . you want that to be a personal term as far as instructor is more formal [Participant 13, FG1, Round 2]
Motivational accountability	I think it’s motivational to see what your activity is. I mean that’s why I log my miles . . . we keep a sheet. And we just kind of record it so we do know something. It’s not like we go back and look but at least you could say oh look we went two weeks here . . . I mean its accountability. And if you have accountability, I think that kind of is a motivator . . . [Participant 4, FG1, Round 1]
	The coach does make you accountable. I mean, when you know someone’s calling you, I have to give my feedback which commits me a little more. When I have to verbalise something, I think or write it down [Participant 16, FG1, Round 2]
	I like the contract concept and meeting the person one-on-one . . . both of you have responsibility, it’s not just on me, it’s on you too, the therapist or the counsellor, it’s a two-way street [Participant 12, FG2, Round 2]
Motivational education	Let’s see the studies [Participant 18, FG1, Round 1]
	. . . want to know that what I’m doing has reasonable logical sense in direction [Participant 20, FG2, Round 1]
	I want to know that you guys have had some research that showed achievable, quantifiable results [Participant 7, FG2, Round 1]

**Table 2.** Theme 2 – barriers to engagement with the intervention.

Sub-theme	Participant quote
Suboptimal interaction with healthcare professionals	To me if you know they are saying oh, you are doing so well, and it’s in their voice ‘for someone your age’, ‘well, I’m so proud you are getting around’, that kind of stuff, yeah, it really ticks me off [Participant 8, FG2, Round 1]
	I call them the ‘I know’ statements like well you know, you need to do this it’s like yeah, I know like what’s the use of you even saying that to me it just makes you feel bad that I didn’t do it and maybe ask why it’s like what because I don’t look forward to the pain that ensues once I tried to do this whatever it is . . . I just didn’t want to hear it from her anymore [Participant 9, FG1, Round 2]
	Oh, she’s going to call me . . . I’m going to have to make something up. . . There is definitely a trust thing, it’s definitely just like I said, with a therapist there; you have to create that bond of trust between the two of you otherwise, it’s not going to work, it just really doesn’t work [Participant 8, FG2, Round 2]
Recording behaviour as burdensome/disruptive	Way too much detail in the log – more detail and nobody’s going to do in the log like [Participant 11, FG1, Round 2]
	Gets overwhelming where it’s like, I’m going to throw up my hand and say, screw it. Forget it. I’m not doing this [Participant 6, FG1, Round 2]
	Well I’ve been in several studies where I had to do that. And every one of them had this giant honking thing on my arm and it interfered with everything I wanted to do. It was huge. It was uncomfortable . . . [Participant 18, FG1, Round 1]
Uncertainty about technique	It’s hard to describe to people over the phone . . . even if it’s a little video conference . . . they can very quickly just spot something that you’re doing physically and go, oh, you know. When you do that that might actually cause more pain. They can’t see that on a telephone call at all [Participant 3, FG1, Round 1]
	if I’m trying to communicate with somebody, a lot of times, if I can’t read their face in response to what I’m saying, we’re going to have missteps no matter what [Participant 10, FG2, Round 2]
	I like knowing that somebody has assessed what I can do and what I can’t do and what I can move towards [Participant 12, FG2, Round 1]

**Table 3.** Theme 3 – characteristics of the physical activity component.

Sub-theme	Participant quote
Tailored	I like a structured class, I do structured classes now and it’s for the socialise, especially with the benefit of being retired [Participant 10, FG2, Round 1]
	I’m more of an outside person, I get out . . . I go down to the river walk and I walk [Participant 9, FG2, Round 1]
	I think you need choices because everybody around the table is – I’m hearing different things [Participant 15, FG1, Round 2]
Sustainable	To me sustainability is the thing [Participant 19, FG2, Round 2]
	This is progressing with something you’ve established . . . and maintaining that [Participant 10, FG2, Round 2]
	I had a PM&R [Physical Medicine and Rehabilitation] regimen that was like six hours a day exercises and I said you got to be kidding me yeah, I’m in pain but I have a life too [Participant 6, FG1, Round 2]
Supported	The coach could help you and give you new ideas or a new way of doing something if you’re stuck because you’re looking for progress [Participant 2, FG1, Round 2]
	I was thinking, also something about the coach the person you’re exchanging information with, for them to ask questions, not just about having been able to do this or meeting your goal, but kind of like bigger picture questions like how are you feeling about this overall [Participant 11, FG1, Round 2]
	as you get older you get distance from a lot of people so that human contact too is somebody cares about me if I’m healthy or not [Participant 3, FG1, Round 2]

## Facilitators of engagement with the intervention

Motivation to uptake and engage in the intervention was an overarching factor within a ‘facilitators’ theme. This could be differentiated into sub-themes that described the motivational power of language, being held accountable and being educated about potential benefits.

### Motivational language

Participants described the motivational power of language to encourage actions/behaviours while avoiding loaded terms associated with previous negative experiences. This was most evident for the use of the word ‘exercise’, which evoked childhood memories of school punishment. ‘Move’, ‘movement’ or ‘motion’ were discussed as more acceptable, as was ‘physical activity’, although the latter was perceived to be related to everyday activities and not a structured time for self-care. There were fewer negative associations with the word ‘sleep’. However, there was some diversity, with participants with particularly severe sleep problems explaining that the word aroused feelings of anxiety or guilt. More acceptable words included ‘snooze’, ‘rest’ and ‘recover’, as well as references to ‘bed’. The use of slogans (both existing, e.g. ‘motion is lotion’, as well as those co-produced during the focus groups, e.g. ‘snooze, move, improve’) were described as helpful motivators that could be included in a companion intervention workbook. Language was also considered important when describing healthcare providers, with the term ‘coach’ preferred to more formal titles or those that referred to clinical roles which were perceived to possess a disciplinary dimension.

### Motivational accountability

Specific methods to support motivation were raised, with being held accountable in some way identified as key. Different perspectives on what it might mean to be held accountable were discussed, with both *keeping* a record of activities (writing down goals; physically ticking them off) and *sharing* a record or being observed by others (healthcare professionals or friends/family members) being valued motivators. The requirement to enter nightly sleep data (e.g. number and duration of awakenings) into the *Sleepio* programme was perceived as sufficient to support self-accountability to the sleep component, but a record of accountability for continued engagement in physical activity was also considered essential. Self-monitoring using an intervention workbook was agreed as a helpful way to keep oneself accountable to physical activity goals. This was also identified as a helpful tool to keep a written record of sleep, avoiding any anxiety that might arise as a result of having to remember times of awakening or having to turn on electronic devices to enter data into the *Sleepio* interface straight away. Different sources of external accountability were identified as having different types of benefit. Reporting on progress to healthcare professionals (coaches) was perceived as a motivator by having a witness of, and providing encouragement to, remain committed to the programme. The concept of setting clear expectations and even agreeing on a contract of participation at the start of the programme was raised as a potentially helpful device to support external accountability. Having buddies (friends/family members) to be accountable to was also viewed as helpful and a less formal way to support on-going motivation. It was not perceived to be important that buddies were also participating in the intervention.

### Motivational education

There was agreement that motivation could be enhanced, and scepticism reduced, if evidence that supported the potential for improvements in both sleep and physical activity to reduce pain was provided. Although participants agreed that the holistic nature of the intervention made sense, and the ‘joined-up-ness’ of the approach appreciated, within both groups, there was an appetite for access to research, and explanations about why the intervention components may be effective. This led to suggestions for a section of an intervention workbook to contain clearly described evidence, with easy-to-digest, visually presented information. Although most were familiar, and had previous experience of, the potential benefits of physical activity for pain management, sleep improvement for analgesia was less well known and was identified as important to highlight in any educational content.

## Barriers to engagement with the intervention

Thematic barriers to intervention uptake and continued engagement could be interpreted as ‘shadows’ to facilitator sub-themes of language, accountability and education: suboptimal interaction with healthcare professionals, excessive recording of behaviour and uncertainty about technique.

### Suboptimal interaction with healthcare professionals

Suboptimal communication and interactions with healthcare professionals were identified as potentially demotivating factors, with participants expressing the need for honest and empathic support and feedback, including provision of constructive criticism. Previous experiences of being patronized, underestimated or not feeling cared for were recalled. Interactions with healthcare providers limited to ‘check-box’ exercises, such as use of questionnaires to monitor on-going participation or symptom change were described as cold and clinical, with the potential to instigate a cycle of inauthenticity between participant and coach. Similar experiences in the past were recollected, with participants describing this influencing them to tell healthcare professionals what they wanted to hear so that routine assessments could be completed as quickly as possible. To address this, participants agreed that empathic coaches who asked open-ended questions, actively listening and exploring feelings, were essential.

### Recording behaviour as burdensome/disruptive

Although recording progress was agreed as motivating (facilitator sub-theme: ‘motivational accountability’), excessive self-monitoring was identified as a potential barrier, reducing intervention attractiveness. This was informed by previous experiences using online spreadsheets and paper forms. Those with prior experience of research-grade wearable technology (e.g. wrist-worn actigraphy) from previous studies stated that device design was important. Large, clinical-looking devices were perceived to be burdensome and medicalize the user, with the potential to induce feelings of stigmatization. To mitigate this, the use of streamlined, easy-to-use devices that minimally interfere with daily life was recommended. The burdensomeness of recording was also related to contact with coaches. Although identified as a facilitator (motivational accountability), excessive contact and assessment were identified as demotivating, with the potential to contribute to suboptimal interactions (barrier sub-theme 1).

### Uncertainty about technique

Participants found the online, virtual avatar professor-led nature of the course of dCBT-I attractive. Check-ins (via telephone or Internet) from a coach to support physical activity were discussed and agreed upon as important (weekly agreed as optimal). However, concerns about undertaking movement safely and ‘correctly’ and setting appropriately challenging activity goals were raised. There was a consensus that a thorough, visual assessment at the start of the intervention was important to address uncertainty. It was recognized that different people had different histories of engagement with physical activity, and that this assessment should identify individual preferences, capabilities, necessary adaptations, potential for progression and realistic goals.

## Characteristics of the physical activity component of the intervention

Combining sleep and physical activity improvement in a single intervention was well received. However, the mode and method of delivery of a physical activity component around weekly dCBT-I was open to question. Possible approaches were presented at the first round of focus groups (e.g. structured group class, 1-on-1 with a healthcare professional and telephone-delivered functional goal setting). Perspectives from both groups were shared at Round 2 and discussed further. From these discussions, three sub-themes were developed – the physical activity component should be: (1) tailored to the individual; (2) sustainable beyond the intervention timeline and (3) supported by regular contact/advice from an empathic coach.

### Tailored

No one exercise format was preferred. Some favoured group classes to support accountability and social interaction; others preferred flexibility to undertake exercise when and where it suited them. There was agreement that there was no need to develop a new exercise programme, but that existing and familiar resources could be used. This individualized characteristic was also felt to be necessary given the heterogeneous impact of OA on people’s physical abilities.

### Sustainable

It was recognized that the aim of the intervention was to encourage sustained behaviour change. Most participants had a long history of OA and described participation in physical activity interventions in the past, but described maintenance or progression as challenging. Achievement of realistic activity goals that could be sustained and developed over time was perceived as an ideal outcome. Participants felt that by incorporating familiar and valued activities (e.g. attending a gym or dance class, progressing a walking or jogging practice), opportunities to promote sustained development could be harnessed.

### Supported

Two types of social support for physical activity were identified: social support from healthcare providers (‘coaches’) and from family or friends (‘buddies’). Coaches were perceived as experts who could provide information and motivation (see facilitator sub-theme 2: accountability), review goals, discuss strategies to address any setbacks and help problem solve (e.g. suggest alternative strategies) and encourage reflection on links between sleep, physical activity and pain. The support of buddies was linked to both physical activity accountability (facilitator sub-theme 1) and also as an opportunity to address loneliness. This was particularly pertinent for older members of the group who described a reduction in social contact after retiring from the workplace.

## Discussion

This study explored important aspects of the design and delivery of a hybrid sleep and physical activity improvement intervention from the user perspective. Sub-themes align with and build on previous insights from research focussed on behavioural pain management interventions for people living with OA^4^ and provide practical information that will be used to guide completion of intervention materials and delivery plans.

In accord with previous qualitative research focussed on communicating messages about physical activity,^49^ there was consensus that the language used is fundamentally important to ensure buy-in. Iterative discussions led to the development of possible names for the intervention and associated healthcare professionals. It was agreed that the intervention would be called the *Move & Snooze* programme and healthcare professionals referred to as *coaches*. Identification of accountability (to self and others) as an important motivator has informed the decision to produce a workbook as an intervention companion. Participants agreed that the workbook should include encouraging phrases/quotes and visual summaries of current knowledge. Discussions about the need for regular support (but not too much) resulted in recommendations from group participants for a weekly check-in with a coach over the telephone or Internet. Preferences about communication style were also discussed. Our findings echo those of others in recommending a person-centred, individualized approach that is empathic/compassionate and appreciates intervention participation in the context of the person’s broader life experiences.^50,51^

An important area for exploration was the optimal way of providing a physical activity intervention around a course of dCBT-I. Our sub-themes can be mapped onto behaviour change techniques that have been identified as effective in supporting adoption and/or maintenance of physical activity programmes for adults with long-term conditions:^52^ goal setting and action planning both relate to our sub-themes of ‘tailored’ and ‘supported’ exercise; problem-solving and providing feedback about physical activity/receiving instruction or demonstration of specific physical activities relate to our sub-theme of ‘supported’ exercise and our barrier sub-theme of ‘uncertainty about technique’ and self-monitoring relates to our facilitator sub-theme of ‘accountability’ and our barrier sub-theme of ‘recording behaviour as burdensome’. The importance of tailoring the physical activity component for each individual is also consistent with results from a recent survey.^53^

‘Tailored’ and ‘supported’ themes are also described in a synthesis of qualitative evidence regarding beliefs of people with OA about exercise interventions.^4^ The importance of identifying physical activities that an individual enjoys and that have a social element has also been recommended.^4,53^ Although consistent with intrinsic motivators described within a self-determination theory framework,^54^ we do not plan to incorporate a prescribed social element to the *Move & Snooze* programme as this was not agreed as important by all participants. However, we plan to identify the preferred amount and type of social support for each user and will provide time for discussion about this. We also plan to explore the importance of a social element of the physical activity component further in post-study interviews with those who complete a future feasibility study.

One-to-one weekly contact with a coach was agreed upon as optimal to support continued engagement with the intervention and to provide time for any issues that may arise. Informed by perspectives gathered at the focus groups, this one-to-one coach contact is supported by evidence that suggests that individual consultations will be better attended than group meetings/classes if/when the intervention is offered in a clinical context.^55^ Along with the workbook, these methods situate well with evidence of behaviour change techniques with greatest effectiveness in the short-term, including receiving prompts from therapists and providing materials that can be taken home as an aid.^52^ Behaviour change techniques that have demonstrated effectiveness in the long term include patient-led goal setting^56^ and self-monitoring,^57^ both of which will be integrated in our design.

The use of a ‘behavioural contract’ and ‘non-specific rewards’ has also been identified as particularly useful techniques to support activity behaviour change among adults with OA.^52^ The use of a contract was raised during focus groups to support expectation setting and motivation through being held accountable. Given the fact that the notion of a baseline behavioural contract was raised by a participant, found acceptable by others and that there is strong evidence for its effectiveness in supporting behaviour change, we plan to include a contract at the front of the workbook. This may be particularly beneficial with respect to the sleep restriction aspect of CBT-I, which can present adherence issues^58^ and therefore may be the most difficult aspect without face-to-face therapeutic support. Focus groups did not reveal material rewards as a salient motivating factor. When this was discussed, improvement in symptoms (sleep, pain and/or physical activity) were described as enticing rewards in themselves (e.g. ‘if I’m going to get pain relief and great sleep that’s reward in itself for me’, Participant 6, Group 1, Round 2).

Previously developed hybrid approaches that have incorporated sleep improvement strategies for pain management have focussed exclusively on psychological or educational content and sleep and/or pain outcomes.^21,23–25,59^ Physical activity/exercise is yet to be meaningfully incorporated. However, a protocol for a trial of in-person CBT-I combined with pain neuroscience education for people with chronic spinal pain and comorbid insomnia has been published with physical activity as a secondary outcome (to be measured using actigraphy), alongside pain and sleep measures.^60^ To our knowledge, no other intervention is in development that has a specific and primary focus on the *balance* between sleep and physical activity among an OA population. However, we acknowledge that many exercise interventions include short modules on sleep, albeit usually limited to sleep hygiene education which, when delivered as a sole sleep improvement approach, has been shown to be minimally effective.^42,61,62^

Study strengths include use of a deductive approach, making use of extant theories and evidence, alongside an inductive approach to allow meaning to be developed from discussions regardless of pre-specified domains. Also, the use of repeated focus groups 1 month apart allowed participants to provide initial input and then help refine/amend developed materials. Regarding limitations, it is possible that advertisement respondents had high levels of self-efficacy as they were actively looking for opportunities to participate in research. This may indicate a desire to gain control over symptoms and a tendency to be organized and goal focussed. These qualities arguably relate directly to Bandura’s concept of self-efficacy.^63^ It is therefore possible that the perspectives that we have synthesized are most relevant to those with high symptom management self-efficacy. To address this, when recruiting participants to the feasibility study, we aim to include people with a range of levels of self-efficacy related to use of digital technologies and participation in cognitive behavioural therapy and physical activity. To achieve this, we will review self-efficacy outcome measures and select the most appropriate to incorporate. We also plan to collect data on comorbidities and underlying pain mechanisms that may moderate the effect of the intervention (e.g. fibromyalgia; evidence of nociplastic pain), as well as other moderators and/or mediators of potential effects (e.g. symptoms of anxiety and depression; positive and negative affect; vigilance to pain)^64^. A focus group study design was selected to support rich data collection. However, it is possible that the format, in combination with materials used to stimulate discussion (i.e. the topic guide for Round 1; slideshow for Round 2) could have biased participants to report perspectives that they perceived as ‘preferred’ to appease peers and researchers.^65,66^ This will be borne in mind when one-to-one semi-structured interviews are conducted at the end of the planned feasibility study. Finally, while consideration of cost-effectiveness of the intervention may be premature, promising findings from a recent study of the automated dCBT-I component (cost beneficial when compared with no or other insomnia treatment) support continuing to the planned feasibility study.^67^

## Conclusion

We have gathered perspectives from people living with OA-related pain and sleep disturbances on the design and delivery of a new hybrid sleep and physical activity improvement intervention. Insights will be incorporated into intervention materials and protocols. Feasibility and acceptability of the developed intervention will be assessed in a future feasibility study, after which further qualitative work will be undertaken with the aim of refining the intervention prior to testing its effectiveness in a suitably powered trial.

## Supplemental Material

sj-docx-1-bjp-10.1177_20494637211026049 – Supplemental material for Development of a hybrid sleep and physical activity improvement intervention for adults with osteoarthritis-related pain and sleep disturbance: a focus group study with potential users Click here for additional data file. Supplemental material, sj-docx-1-bjp-10.1177_20494637211026049 for Development of a hybrid sleep and physical activity improvement intervention for adults with osteoarthritis-related pain and sleep disturbance: a focus group study with potential users by Daniel Whibley, Kevin Stelfox, Alasdair L Henry, Nicole KY Tang and Anna L Kratz in British Journal of Pain

sj-docx-2-bjp-10.1177_20494637211026049 – Supplemental material for Development of a hybrid sleep and physical activity improvement intervention for adults with osteoarthritis-related pain and sleep disturbance: a focus group study with potential users Click here for additional data file. Supplemental material, sj-docx-2-bjp-10.1177_20494637211026049 for Development of a hybrid sleep and physical activity improvement intervention for adults with osteoarthritis-related pain and sleep disturbance: a focus group study with potential users by Daniel Whibley, Kevin Stelfox, Alasdair L Henry, Nicole KY Tang and Anna L Kratz in British Journal of Pain
