# Supplementary material for: Development of a hybrid sleep and physical activity improvement intervention for adults with osteoarthritis-related pain and sleep disturbance: a focus group study with potential users
Source: Br J Pain. 2021 Jun 25;16(2):136–48. doi: 10.1177/20494637211026049 (PMC8998527; doi:10.1177/20494637211026049)
Supplement: sj-docx-1-bjp-10.1177_20494637211026049 – Supplemental material for Development of a hybrid sleep and physical activity improvement intervention for adults with osteoarthritis-related pain and sleep disturbance: a focus group study with potential users [file sj-docx-1-bjp-10.1177_20494637211026049.docx]

**Focus Group 1: Topic and question guide**

**Opening Statements**

Thank you for agreeing to participate in this research project. I’m [xx], a researcher in the xxxx at the xxxx and I will be your facilitator for this discussion today.

[Co-facilitator introduces themselves]

Everyone here has been recruited as they live with osteoarthritis-related pain. We’re developing a new intervention that is aiming to reduce OA-related pain by helping people to improve both their sleep and levels of physical activity. The plan is that this will intervention will combine an internet-delivered sleep improvement program with physical therapist-led exercise. The best way to make sure that this new combined intervention is acceptable to patients is to involve the kinds of people who may be offered it to assist in its development. This will help to make it as attractive as possible.

We’ve asked you to meet with us here today for a “focus group”. As you might know, a “focus group” is a group of people that get together to talk about some topic, so that we can learn what they think or know about that topic. In a focus group it’s really important that you say what you think. There are no right or wrong answers. We want to know what *you* think. It’s important that people with different perspectives speak up. If you would like to add to an idea, or if you have an idea that’s different from someone else’s, please feel free to jump in. You don’t need to wait for me to call on you to talk, but only one person should talk at a time.

You have all been invited back to the second focus group in February. At that group we will show you what we have developed based on your feedback today. That is likely to include a prototype copy of a manual that will be given to patients who choose to engage with the intervention.

**Group Ground Rules**

We will be audio recording this session to be sure we don’t miss anything you say, but we won’t be keeping track of who said what, so anything you say is anonymous. That is, your comments will not be linked to your name. Please speak up and talk one at a time so we can hear you clearly when the recording is transcribed. We have 2 hours for the focus group today and it will be my job to will keep us moving along. In focus groups we want the discussion to be among you, because it is *your* thoughts and opinions that we want to hear. We want you to talk with each other and express your experiences and opinions. I will keep the discussion focused on topics we want to know about, so please don’t be offended if I redirect the discussion. I am here to facilitate the discussion, but it’s important that the interaction flows among you without much input from me. I might take some written notes during the group.

**I. Introduction & Ice-Breaker**

Let’s go around the room and briefly introduce ourselves. In a few sentences, tell us your first name and why you decided to participate in the focus group.

**II. Introduce the initial intervention idea**

There is evidence that improving sleep and improving physical activity levels can help reduce pain for people with osteoarthritis. We think it makes sense to use this evidence to develop an intervention that concurrently aims to improve both sleep and physical activity to have the biggest impact on pain.

As a starting point, our intervention consists of:

1) An online course of Cognitive Behavioral Therapy for insomnia. The program we are using is called *Sleepio*. It uses animations, audio and text to deliver a program that can be completed at home over the internet. It has been shown to improve sleep by changing thought, beliefs and behaviors around sleep. The course lasts six weeks, you interact with it once a week.

We want to supplement this 6-week *Sleepio* program with a physical activity intervention. There are a few options regarding how we might do this and we will be discussing this in the group today.

**Topic: Optimal mode of delivery of the exercise intervention**

CBTi will be delivered online – 1 session per week, led through with an animated professor. We are designing the physical activity or exercise component to fit around this and there are a few options.

What do you think of these options?

- Group, supervised exercise class (x1 per week *or* x2 per week)
- 1-to-1 face-to-face exercise versus group exercise class
- Instead of a structured exercise class, telephone-delivered goal setting for personalized functional activity goals

**Topic: Attitudes toward the intervention and motivations to use it; expectations and barriers to completion of a six-week course**

- What is your overall initial impression of the proposed intervention?
- How do you feel about combining/integrating CBTi delivered over the internet with a PT-led physical activity program with time set aside to review progress with the online CBTi course?
- What would motivate you to use it or stick with it?
- Is there anything that would make you more likely to want to use it and stick with it?
- What things do you think might be barriers to using it or sticking with it?
- What do you think about how this would fit into your wider life and life responsibilities?
- What do you think about the time commitment required from the intervention? (six weeks, CBTi once a week, PT-led PA once or twice a week)?
- If this is problematic, how might this be improved?
- For those of you in employment, do you think there are any other barriers?
- How about friends of family?

**Topic: The language we use to promote positive sleep and physical activity/exercise behaviors**

We want to gather information on is the best type of language and messaging to use. We want to make sure that we are using appropriate language to ensure that people who use our intervention remain engaged.

**Sample questions:**

- *Previous research suggests we should avoid the work ‘exercise’ and that ‘physical activity’ should be used instead. What do you think about this?*
- *Sometimes researchers use the term ‘perceived exertion’ to refer to how hard you feel you are working during physical activity. What do you think about this term? [Can you think of any alternatives that you might find more suitable?]*
- *Are there any ways that you do or don’t like sleep to be described or referred to?*
- *Sometimes researchers refer to ‘sleep efficiency’ to describe how much time you spend in bed is actually spent asleep. What do you think about this term? [Can you think of any suitable alternatives?]*

*Additional questions if time permits*

- *Sleep and fatigue are different things. You can consistently get an appropriate amount of sleep and yet still feel fatigue. What do you think about these different yet related experiences?*
- *How are sleep and physical activity related for you? How do they relate to your pain?*
- *We often ask about four types of sleep disturbance: trouble getting to sleep, staying asleep, waking up earlier than you’d like, and feeling refreshed on awakening. We’d like to explore what the last one, feeling ‘refreshed upon awakening’ feels like or means to you. What is your experience of this? [Follow-up question… how does this sense of ‘unrefreshed sleep’ relate to pain and activity levels for you, particularly in the morning?]*

**Topic: Motivational feedback**

We are developing a manual and are likely to include motivational messages or feedback. We might also provide motivation over email or text message during the program.

- With regard to physical activity, what kind of messages do you think you would find motivating to support changing your behaviors?
- What kind of messages do you think would discourage you or ‘turn you off’?
- With regard to sleep behaviors – like keeping a regular sleep routine, or changing what you do at bedtime - what kind of messages do you think you would find motivating to support changing your behaviors?
- For sleep behaviors, what kind of messages do you think would discourage you or ‘turn you off’?
- What do you think about receiving encouraging or reminding messages?
- What would these ideally look or sound like to you?

**Topic: Receiving progress reports and results**

The intervention lasts six weeks, and there will be follow-up with people 3 months after they first start

- What do you think about receiving personalized progress reports during the intervention?
- Would this motivate you or would this be an overload of information?
- People will be asked to wear watch-like devices so that we can measure sleep and physical activity. What do you think about this? Are you interested in detailed readouts from these devices?
- How would you feel about receiving feedback via emails or text messages?

**Topic: Social interaction during the intervention**

- Is interacting with other people who are using the intervention important for you?
- Is face-to-face interaction important?
- Is online interaction (e.g. some kind of chatroom) appealing?

**Focus group closure**

- Did the topics today bring to mind any other issues that you think we should be thinking about?
- Any questions from the co-facilitator?

Thank you very much for your participation today. I will be in touch with you all to arrange attendance at the next group in February.
