# Supplementary material for: Development of a hybrid sleep and physical activity improvement intervention for adults with osteoarthritis-related pain and sleep disturbance: a focus group study with potential users
Source: Br J Pain. 2021 Jun 25;16(2):136–48. doi: 10.1177/20494637211026049 (PMC8998527; doi:10.1177/20494637211026049)
Supplement: sj-docx-2-bjp-10.1177_20494637211026049 – Supplemental material for Development of a hybrid sleep and physical activity improvement intervention for adults with osteoarthritis-related pain and sleep disturbance: a focus group study with potential users [file sj-docx-2-bjp-10.1177_20494637211026049.docx]

**Supplement 2. Themes, sub-themes, and participant quotes**

| **Theme** | **Sub-theme** | **Participant quote** |  |
| --- | --- | --- | --- |
| **Facilitators of engagement with the intervention** | Motivational language | When I was a kid, when were in P.E. class and we did something wrong we had to run an extra lap or work out extra was like punishment, but it should be a gift like health enhancement activities or something… It's got a bad taste in my mouth, ‘exercise’ [Participant 3, FG 1, round 1] |  |
|  |  | Yeah, ‘exercise’ to me just like a punishment, I have very negative feelings about ‘exercise’, I don’t like it [Participant 8, FG 2, round 1] |  |
|  |  | I really like ‘move’ because I have this real negative connotation with ‘exercise’… ‘physical activity’ is better or ‘move’ in this context is really good [Participant 6, FG1, Round 2] |  |
|  |  | good to have little mantras and I think the little kind of things you tell yourself and keep you with that little exponent or something [Participant 4, FG2, Round 2] |  |
|  |  | I like the ‘move, recover and progress’ because in fitness, you have to take time down to recover to let your muscles recover, you can't keep doing the same thing… I like ‘move recover, progress’ because it shows forward movement [Participant 8, FG2, Round 1] |  |
|  |  | if you're in a sport in school and you have a coach, they're as bigger cheerleader to you as what your football cheerleading team would be. So your coach is kind of… you want that to be a personal term as far as instructor is more formal [Participant 13, FG1, Round 2] |  |
|  |  | It’s not punitive… coach is more neutral, more supporting [Participant 6, FG1, Round 2] |  |
|  | Motivational accountability | I think it's motivational to see what your activity is. I mean that's why I log my miles… we keep a sheet. And we just kind of record it so we do know something. It's not like we go back and look but at least you could say oh look we went two weeks here… I mean its accountability. And if you have accountability, I think that kind of is a motivator… [Participant 4, FG1, Round 1] |  |
|  |  | I usually use my phone. I just started a little thing that's like a habit tracker because I'm trying to discipline myself to be a more scheduled structured human being because I'm very chaotic in my life…. when I check it off, I feel like I actually accomplished it. More so than knowing that I just did it and didn’t mark anything down on my list [Participant 13, FG1, Round 1] |  |
|  |  | having some sort of tracker like that is really actually very helpful because at a glance you see yep did that. And it also gives you that little reward [Participant 4, FG2, Round 2] |  |
|  |  | I think writing it down is easier because you do it at the moment. And then you can always transfer it digitally [Participant 2, FG1, Round 1] |  |
|  |  | I'm going to write it down anyway before going to put it online [Participant 18, FG1, Round 1] |  |
|  |  | the coach does make you accountable. I mean, when you know someone's calling you, I have to give my feedback which commits me a little more. When I have to verbalise something, I think or write it down  [Participant 16, FG1, Round 2] |  |
|  |  | Like buddy seems more like check-in but people running the study would seem better for staying on track and encouraging and for me, just for me, phone calls [Participant 4, FG1, Round 1] |  |
|  |  | [a written contract] would make me less anxious, because it's not as nebulous, it's not as out there [Participant 8, FG2, Round 2] |  |
|  |  | I like the contract concept and meeting the person one-on-one… both of you have responsibility, it's not just on me, it's on you too, the therapist or the counsellor, it's a two-way street  [Participant 12, FG2, Round 2] |  |
|  |  | [a buddy] could be anybody because I mean if I did it with just a friend or even like my mom or somebody even if she's not in the program and doesn't have the same issues as me… It's a support system that a lot of people just need it [Participant 13, FG1, Round 1] |  |
|  | Motivational education | Let's see the studies [Participant 18, FG1, Round 1] |  |
|  |  | …want to know that what I'm doing has reasonable logical sense in direction [Participant 20, FG2, Round 1] |  |
|  |  | I want to know that you guys have had some research that showed achievable, quantifiable results [Participant 7, FG2, Round 1] |  |
|  |  | the older I get more I feel I'm just overwhelmed with data and information and words in general. Yeah. And things that are translated into visuals you can look at a quick, okay, yeah moments instead of reading three paragraphs and tell me that I have to get up to this level by week two [Participant 3, FG1, Round 2] |  |
|  | | | |
| **Barriers to engagement with the intervention** | Suboptimal interaction with healthcare professionals | To me if you know they are saying oh, you are doing so well, and it's in their voice ‘for someone your age’, ‘well, I'm so proud you are getting around’, that kind of stuff, yeah, it really ticks me off [Participant 8, FG2, Round 1] |  |
|  |  | I call them the “I know” statements like well you know, you need to do this it's like yeah, I know like what's the use of you even saying that to me it just makes you feel bad that I didn't do it and maybe ask why it's like what because I don't look forward to the pain that ensues once I tried to do this whatever it is… I just didn’t want to hear it from her anymore [Participant 9, FG1, Round 2] |  |
|  |  | if I just think that you're on my team and you make me feel like you care, I'm all in. But if I feel like you're barking at me, I'm all out, man [Participant 11, FG1, Round 1] |  |
|  |  | …just flat out lie and go oh yeah, I did those last week or say, you know I don't have the time or I was busy or you know when I was asleep and that kind of thing [Participant 3, FG1, Round 1] |  |
|  |  | Oh, she's going to call me… I'm going to have to make something up… There is definitely a trust thing, it's definitely just like I said, with a therapist there; you have to create that bond of trust between the two of you otherwise, it's not going to work, it just really doesn't work [Participant 8, FG2, Round 2] |  |
|  |  | We went through three instructors before we finally hit on one that was more sympathetico… the objection to the first two was the fact that they were simply didn’t relate to the demographic they were working with [Participant 5, FG1, Round 2] |  |
|  |  | If you got feedback with your well, how you are doing with it, rather than it being one way where I absorb it or box check it to have encourage, especially you weren’t an exerciser I wasn’t doing it and I wanted to get started that feedback would be important [Participant 12, FG2, Round 1] |  |
|  | Recording behaviour as burdensome/disruptive | Way too much detail in the log -- more detail and nobody's going to do in the log like [Participant 11, FG1, Round 2] |  |
|  |  | gets overwhelming where it's like, I’m going to throw up my hand and say, screw it. Forget it. I'm not doing this [Participant 6, FG1, Round 2] |  |
|  |  | I was reasonably technologically literate. But it was an operating system that I didn't know. And I was always screwing up. And I'm sure they hated me by the end of it because I just couldn't make it work properly. I kept having to call, having to talk me through doing this then other thing. It's just too much [Participant 2, FG1, Round 1] |  |
|  |  | Well I've been in several studies where I had to do that. And every one of them had this giant honking thing on my arm and it interfered with everything I wanted to do. It was huge. It was uncomfortable… [Participant 18, FG1, Round 1] |  |
|  | Uncertainty about technique | it's hard to describe to people over the phone… even if it's a little video conference... they can very quickly just spot something that you're doing physically and go, oh, you know. When you do that that might actually cause more pain. They can't see that on a telephone call at all [Participant 3, FG1, Round 1] |  |
|  |  | if I'm trying to communicate with somebody, a lot of times, if I can't read their face in response to what I'm saying, we're going to have missteps no matter what [Participant 10, FG2, Round 2] |  |
|  |  | I like knowing that somebody has assessed what I can do and what I can't do and what I can move towards [Participant 12, FG2, Round 1] |  |
|  | | | |
| **Characteristics of the physical activity component** | Tailored | We tend to shy away from that group classes because there's a lot of things that I specifically said I cannot do. And they're running the whole class along. And I said and thinking you got to be kidding me. I can't do that. There's just no possible way. And now I feel weird because other people look at me like what's wrong with her. Why isn't trying kind of thing? [Participant 4, FG1, Round 1] |  |
|  |  | I like a structured class, I do structured classes now and it's for the socialise, especially with the benefit of being retired [Participant 10, FG2, Round 1] |  |
|  |  | now that we are retired want to do things on our own time, on our own schedule, on our own agenda. And so, for me just locking me into saying Tuesdays at eight o’clock in the morning you will be doing da-da-da would not be very attractive. I like to have more agency about that [Participant 20, FG2, Round 1] |  |
|  |  | I'm more of an outside person, I get out… I go down to the river walk and I walk [Participant 9, FG2, Round 1] |  |
|  |  | I think you need choices because everybody around the table is – I'm hearing different things [Participant 15, FG1, Round 2] |  |
|  | Sustainable | To me sustainability is the thing [Participant 19, FG2, Round 2] |  |
|  |  | This is progressing with something you’ve established… and maintaining that [Participant 10, FG2, Round 2] |  |
|  |  | I had a PM&R [Physical Medicine and Rehabilitation] regimen that was like six hours a day exercises and I said you got to be kidding me yeah, I'm in pain but I have a life too [Participant 6, FG1, Round 2] |  |
|  |  | My goal… to keep what I had, and to do things that are easy to do and if I get so that they’re too easy to do, then I can do a little bit more, but I'm not going to do you know… I'm looking to keep what I got and I've got arthritis there's nothing you're going to change about that… all I want to do is something that keeps me going so it doesn't get worse [Participant 3, FG1, Round 2] |  |
|  | Supported | Sometimes they kind of like there is someone who feels I can do more than I think I can do because sometimes I underestimate on those views and someone will say well, you know if you can do this, I think you can do this. And have coaches into thinking yeah, good, if I can get a little success [Participant 12, FG2, Round 2] |  |
|  |  | if you're stuck, the coach could help you and give you new ideas or a new way of doing something if you're stuck because you're looking for progress, right so [Participant 2, FG1, Round 2] |  |
|  |  | I was thinking, also something about the coach the person you're exchanging information with, for them to ask questions, not just about having been able to do this or meeting your goal, but kind of like bigger picture questions like how are you feeling about this overall [Participant 11, FG1, Round 2] |  |
|  |  | this pain stuff, the aloneness, the isolation, this is what makes me just want to kind of give up and not do anything [Participant 11, FG1, Round 1] |  |
|  |  | as you get older you get distance from a lot of people so that human contact too is somebody cares about me if I'm healthy or not [Participant 3, FG1, Round 2] |  |
|  | | | |
